# Efficacy of orally administered fluralaner (Bravecto^TM^) or topically applied imidacloprid/moxidectin (Advocate®) against generalized demodicosis in dogs

**DOI:** 10.1186/s13071-015-0775-8

**Published:** 2015-03-28

**Authors:** Josephus J Fourie, Julian E Liebenberg, Ivan G Horak, Janina Taenzler, Anja R Heckeroth, Regis Frénais

**Affiliations:** ClinVet International, Uitsigweg, Bainsvlei, 9338 Bloemfontein, Free State South Africa; Department of Veterinary Tropical Diseases, Faculty of Veterinary Science, University of Pretoria, Onderstepoort, 0110 Pretoria, South Africa; MSD Animal Health Innovation GmbH, Zur Propstei, 55270 Schwabenheim, Germany; MSD Animal Health Innovation SAS, 49071 Beaucouzé Cedex, France

**Keywords:** Bravecto™, Chewable tablets, Fluralaner, Advocate®, Spot-on, Imidacloprid, Moxidectin, Efficacy, Dogs, Generalized demodicosis, *Demodex canis*, Mange

## Abstract

**Background:**

This laboratory study compared the efficacy of Bravecto™ (fluralaner), formulated as a chewable tablet, with the efficacy of Advocate® (imidacloprid/moxidectin), formulated for topical administration, against naturally acquired generalized demodicosis in dogs.

**Methods:**

Sixteen dogs, all diagnosed with generalized demodectic mange, were randomly allocated to two equal groups. Bravecto™ chewable tablets were administered once orally at a minimum dose of 25 mg fluralaner/kg body weight to one group of dogs, while the second group was treated topically on three occasions at 28-day intervals with Advocate® at a minimum dose of 10 mg imidacloprid/kg body weight and 2.5 mg moxidectin/kg body weight. Mites were counted in skin scrapings and demodectic lesions were evaluated on each dog before treatment and at 28-day intervals thereafter over a 12 week study period. Deep skin scrapings (~4 cm^2^) were made from the same five sites on each dog at each subsequent examination.

**Results:**

After single oral administration of Bravecto™ chewable tablets, mite numbers in skin scrapings were reduced by 99.8% on Day 28 and by 100% on Days 56 and 84. Mite numbers in the dogs treated topically on three occasions at 28-day intervals with Advocate® were reduced by 98.0% on Day 28, by 96.5% on Day 56 and by 94.7% on Day 84. Statistically significantly (*P* ≤ 0.05) fewer mites were found on Days 56 and 84 on the Bravecto™ treated dogs compared to Advocate® treated dogs. A marked decrease was observed in the occurrence of erythematous patches, crusts, casts and scales in the dogs treated with Bravecto™ and in the occurrence of erythematous patches in the dogs treated with Advocate®. With the exception of one dog in each treated group, all dogs exhibited hair regrowth ≥ 90% at the end of the study in comparison with their hair-coat at study start.

**Conclusions:**

Single oral administration of Bravecto™ chewable tablets is highly effective against generalized demodicosis, with no mites detectable at 56 and 84 days following treatment. In comparison, Advocate®, administered three times at 28-day intervals, is also highly effective against generalized demodicosis, but most dogs still harboured mites at all assessment time points. Both treatments resulted in a marked reduction of skin lesions and increase of hair re-growth 12 weeks after the initial treatment.

## Background

Historically the only follicular mite considered to infest dogs is *Demodex canis* [1], but two other mites have subsequently been described. Desch and Hillier [2] described *Demodex injai*, a mite that is considerably longer than *D. canis*, while Tamura et al. [3] described a mite that is considerably shorter than *D. canis*, but failed to name it. In the literature this mite is now usually referred to as *Demodex cornei*. The three species differ in length as described by Izdebska and Fryderyk [4] with reference to average values. For *D. canis* adult female mites measured 226.0 μm and the males 195.2 μm. Adult female *D. injai* measured 330.9 μm and the males 371.8 μm. For *D. cornei* adult female mites measured 139.4 μm and the males 120.8 μm. Various molecular studies have been carried out to determine the specific identities of the three mites. Based on the results obtained from the mitochondrial markers 16S rDNA and Cytochrome Oxidase I (COI) for the three morphotypes of *D. canis*, De Rojas et al. [5] concluded that they are polymorphs of the same species. On the other hand phylogenetic analysis of the three species, based on partial sequences of mitochondrial 16S rDNA, led Sastre et al. [6] to propose that *D. canis* and *D. injai* are valid separate species and *D. cornei* a morphological variant of *D. canis*. A year later similar analyses performed by Milosevic et al. [7] confirmed that *D. injai* is a valid species. It is clear that further studies are required to ascertain the specific status of the mite referred to as *D. cornei*.

*Demodex* spp. mites (*D. canis*, *D. injai*, and *D. cornei*) are normal commensals of the dog’s skin parasitising within the sebaceous glands connected to the hair follicles. Should their numbers increase dramatically, they are capable of producing a disease known as canine demodicosis or demodectic mange. Canine demodicosis is an inflammatory parasitic skin disease that can be classified as localized or generalized according to the extent of the lesions. Localized demodicosis occurs as only small areas of alopecia, most commonly on the face and the forelegs. It is a benign disease and most cases resolve spontaneously within six to eight weeks. Demodicosis is considered generalized when five or more areas on the body are affected, or pododemodicosis is observed on two or more feet, or when an entire body region is involved [8,9]. Demodicosis can also be categorized as either juvenile (dogs up to 18 months of age), adult onset (dogs generally older than four years of age with no previous history of disease), or chronic generalized (persisting disease for at least six months) [10,11]. Susceptibility of dogs to infestations with *Demodex* spp. and to progression of the clinical disease are influenced by numerous factors including (listed in decreasing order of importance): immune status which may be affected by debilitating diseases, endoparasitism, breed, age as well as nutritional or hormonal status, other immunological disorders such as genetic defects and alteration of the skin’s biochemistry and structure [12]. Although *D. canis* remains the most common species/morphotype encountered in demodicosis, moderate alopecia, and greasy seborrhoeic dermatitis along the dorsal trunk of dogs has been associated with increased numbers of *D. injai* [13]. Moreover, bilateral ceruminous otitis in a beagle, of which *D. injai* was the cause, has been described [7]. *D. cornei* would appear to be an inhabitant of the host’s stratum corneum [5], and Shipstone [11] mentions that *D. cornei* has been found in association with a generalised, pruritic, scaly dermatitis, with the mites present in the surface scale.

Chronic generalized demodicosis is a frustrating and difficult skin disease to treat [14-16]. In dogs that are otherwise healthy, the generalized form of the disease is unlikely to resolve without therapy [10]. Therapeutic options that are currently available include amitraz, ivermectin, milbemycin oxime and moxidectin, mostly to be given at multiple occasions for periods of three months or more [16-18]. To be effective, these treatment regimens require high owner compliance over an extended period of time.

The active ingredient of Bravecto™, fluralaner, is a novel, long-acting systemic insecticide and acaricide belonging to the isoxazoline class of parasiticides with selective inhibition of arthropod γ–aminobutyric acid- and L–glutamate-gated chloride channels [19]. In field studies involving naturally infested dogs, a single oral administration of Bravecto™, formulated as a chewable tablet, proved to be > 99% effective against fleas and ticks at each measured time-point over a period of 12 weeks following treatment [20]. The dogs in these studies were variably infested with at least four tick species, including ticks belonging to the *Rhipicephalus sanguineu*s group, *Ixodes hexagonus*, *Ixodes ricinus* and *Dermacentor reticulatus*. In a simulated home environment a single oral administration of Bravecto™ formulated as chewable tablets was > 99% effective against the flea *Ctenocephalides felis*, at every time-point measured over a period of 12 weeks following treatment [21]. In a laboratory study designed to detect the speed of kill of Bravecto™ chewable tablets against laboratory infestations of the tick *I. ricinus* on dogs, acaricidal efficacy was 89.6% at 4 hours, 97.9% at 8 hours and 100% at 12 and 24 hours after treatment [22]. An even faster onset of killing activity starting at 1 hour after oral administration of Bravecto™ chewable tablets was observed in dogs experimentally infested with *C. felis* flea [23]. The aim of the current study was to determine a) the efficacy of fluralaner against *Demodex* spp. mites and b) to show efficacy duration over 12 weeks following single oral treatment with Bravecto™ chewable tablets. As positive control, a second group of dogs was treated topically three times at a 28-day interval with Advocate® (imidacloprid and moxidectin).

## Methods

### Study design

In the present study, Bravecto™ administered as chewable tablets on a single occasion was the test product and Advocate®, administered three times at 28-day intervals was included as a positive control. This made it possible to compare the long term efficacy of 12 weeks of a single oral treatment with Bravecto™ chewable tablets against the efficacy of Advocate® administered at a 28-day interval according to the product label.

The study was designed as a parallel group, blinded, randomized, single centre, and positive controlled efficacy study. The study was conducted in accordance with the FDA Code of Federal Regulations: Good Laboratory Practice for Nonclinical Laboratory Studies 2009 [24], and all procedures were in compliance with South African National Standard “SANS 10386:2008: The care and use of animals for scientific purposes” [25]. The protocol was submitted to the ClinVet animal ethics committee that authorized the conduct of the study.

The test system was the individual dog. Dogs with natural infestations of *Demodex* spp. mites and presenting clinical signs of generalized demodicosis, e.g. erythema, hair loss, comedones, follicular casts and crusts were enrolled, with consent from their owners, in the study and were returned to their owners on completion of the animal phase. Dogs included in the study were mostly mongrels and of both sexes, older than 12 months, weighed between 3.5 and 13.7 kg, and except for clinical signs of generalized demodicosis, the dogs were healthy and as far as could be determined the dogs had not been treated with a glucocorticoid or any product with a miticidal effect for at least 12 weeks prior to inclusion. Additional requirements for inclusion were that deep skin scrapings performed before treatment had to be positive for *Demodex* spp. mites.

Sixteen dogs (7 male and 9 female), ranked within sex in descending order of individual pre-treatment mite counts were included in the study and allocated to two equal groups. Each dog was housed individually for the duration of the study in an indoor/outdoor run, without contact between animals, and was fed once a day according to the food manufacturer’s recommendations. Potable municipal water was available *ad libitum*.

Each dog was acclimatized to the housing and maintenance conditions for at least 14 days before treatment. As a precautionary measure all dogs were treated subcutaneously with an antibiotic (cefovecin), appropriate for the treatment of pyoderma on Days −14, −1, 13 and 27. Additionally, on Days −14 and 27, deep skin biopsies were taken from each dog after sedation. The biopsies indicated that exudative pyoderma was present in two dogs in each group on Day −14 and that it had cleared by Day 27. Chronic dermatitis, epidermal acanthosis and hyperkeratosis was present and unchanged in all dogs on both occasions. No inflammatory cells or bacteria were observed in the Day 27 biopsies and antimicrobial therapy was discontinued. Twice during acclimatization (Day −14 and Day −1) and on Days 27/28, 56 and 84 after treatment each dog was clinically examined by a veterinarian.

The dogs were weighed on a calibrated and verified electronic scale on Days −2, 13, 27, 41, 55, 69 and 84 for dose calculation for treatment, for the use of sedatives for skin scrapings and to document the body weight during the study period. General health observations were performed daily throughout the complete study period.

### Treatment

On Day 0, dogs of one group were treated once orally with Bravecto™ chewable tablets (fluralaner, 13.64% w/w), based on the dog’s individual body weight, to achieve a minimum dose of 25 mg/kg body weight and an efficacy over 12 weeks following treatment. The tablet(s) was (were) administered 20 (±10) minutes after food had been offered by placement in the back of the oral cavity over the tongue to initiate swallowing. Also on Day 0, commercially available Advocate® (imidacloprid, 10%/moxidectin, 2.5% w/v) was administered topically to the other group of dogs (positive controls) according to the product label. Due to the 28 days efficacy duration of Advocate®, these dogs were re-treated on Day 28 and 56. With the dog in a standing position, the coat was parted until the skin was visible and the Advocate® was administered directly onto the skin. Both treated groups were observed prior to treatment and again hourly for 4 hours after treatment of the last animal, for possible adverse events. Personnel performing the post-treatment observations were blinded with respect to the treatment.

### Mite assessments

Deep skin scrapings (~4 cm^2^), in which the skin was squeezed and scraped until capillary oozing was seen, were made from five sites on each dog on Days −4, 28, 56 and 84. Skin scrapings of the dogs treated with Advocate® were performed on Day 28 and Day 56, before the second or third treatment was applied, respectively. The same sites and/or sites of new lesions were scraped at each subsequent examination. Each scraping was transferred to a separate labelled microscope slide containing mineral oil and was examined under a stereomicroscope for the presence of *Demodex* spp. mites. The numbers of mites counted in each scraping were recorded separately.

### Skin and hair assessments

The clinical signs and the extent of demodectic lesions on each dog were assessed on the days when skin scrapings were made, and recorded on a standardised form. The following parameters were assessed and sketched on a silhouette (left and right hand side) for each dog: body areas exhibiting erythema; body areas covered by casts, scales and crusts; body areas with hair loss. A semi-quantitative assessment of hair re-growth was performed, comparing hair coat before, within and after the 12 weeks study duration and assessed as percentage hair re-growth (0-50%, 50-90% or > 90%) defined as estimated percentage of hair cover growth compared to baseline total hairless area as assessed prior to veterinary product administration. Colour photographs illustrating the extent of lesions and their resolution were taken of each dog on Day −4 and subsequently at approximately monthly intervals up to Day 84 after treatment.

### Efficacy evaluation

The primary assessment variable in the study was the decrease in total number of mites counted in skin scrapings following treatment.

Efficacy was calculated using geometric means with Abbott’s formula:

$$ \mathrm{Efficacy}\ \left(\%\right) = \left(\mathrm{Mpre}\ \hbox{--}\ \mathrm{Mpost}\right)/\ \mathrm{Mpre}\ \mathrm{x}\ 100 $$

where Mpre was the mean number of pre-treatment mite counts, and Mpost the mean number of post-treatment mite counts.

Additionally, the groups were compared using an ANOVA with a treatment effect after a logarithmic transformation on the mite (count + 1) data, for each study day.

On Day 50 one of the dogs in the group treated with Advocate® developed severe oedema of the left hind leg and prepuce and the swelling did not dissipate with antibiotic, diuretic and corticosteroid treatment. The dog was removed from the study on Day 59, sedated and upon laparotomy a large nodular tumour compatible with malignant lymphoma was found attached to the ventral spine, and the dog was euthanised during surgery. The results pertaining to this dog until Day 56, before its exclusion from the study on Day 59, have been included with those of the other dogs in the group treated with Advocate®.

## Results

No adverse event considered to be related to oral treatment with Bravecto™ chewable tablets or topical treatment with Advocate® was observed in any dog.

Treatment with Bravecto™ chewable tablets resulted in a reduction of the mean mite number present in skin scrapings of 99.8% on Day 28, and of 100% on Days 56 and 84 after treatment. The treatment with Advocate® resulted in a reduction of the mean mite number present in skin scrapings of 98.0% on Day 28, of 96.4% on Day 56, and of 94.7% on Day 84. Statistically significantly (*P* ≤ 0.05) fewer mites were found on the Bravecto™ treated dogs compared to Advocate® treated dogs (Table 1).

**Table 1:** **Geometric mean reductions in** ***Demodex*** **spp. mite counts of dogs treated once orally with Bravecto** **™** **or topically on three occasions at 28-day intervals with Advocate®**

**Study days**	**Study groups**	**Bravecto** **™**	**Advocate®**	**p-value**
**−4**	Mean^a^ mite counts (n)	447.0	509.4/478.6^b^	Na^c^
	Count range (n)	41-1740	79-2724	
**28**	Mean^a^ mite counts (n)	0.8	10.0	
	Count range (n)	0-14	0-496	
	**Efficacy (%)**	**99.8**	**98.0**	**0.0917**
**56**	Mean^a^ mite counts (n)	0.0	18.5	
	Count range (n)	Na^c^	0-115	
	**Efficacy (%)**	**100.0**	**96.4**	**<0.0001**
**84**	Mean^a^ mite counts (n)	0.0	25.6	
	Count range (n)	Na^c^	0-286	
	**Efficacy (%)**	**100.0**	**94.7**	**0.0020**

^a^Geometric mean.

^b^Mite counts calculated without one dog, which was euthanized on Day 59.

^c^Not applicable.

The occurrence of erythematous patches on the dogs treated once orally with Bravecto™ chewable tablets was reduced from 62.5% (5/8) of the dogs on Day −4 prior to treatment to 12.5% (1/8) of the dogs 12 weeks following initiation of treatment. The occurrence of crusts, casts or scales was reduced from 100% (8/8) prior to treatment to 12.5% (1/8) 12 weeks following initiation of treatment. In comparison, the occurrence of erythematous patches on dogs treated three times at a 28-day interval with Advocate® was reduced from 87.5% (7/8) to 0% (0/8) and the occurrence of crusts, casts and scales was reduced from 100% (8/8) to 42.9% (3/7) (Table 2).

**Table 2:** **Reduction in the occurrence of dermatologic changes in dogs with generalized demodicosis after treatment with either Bravecto**™ **or Advocate®**

**Bravecto** **™** **: occurrence of lesions on days before and after treatment (dogs were treated on Day 0: number of dogs/number of dogs per group)**				
**Clinical sign**	**Day −4**	**Day 28**	**Day 56**	**Day 84**
Erythematous patches	62.5% (5/8)	37.5% (3/8)	12.5% (1/8)	12.5% (1/8)
Crusts, casts or scales	100% (8/8)	62.5% (5/8)	62.5% (5/8)	12.5% (1/8)
**Advocate®; occurrence of lesions on days before and after initial treatment (dogs were treated on Days 0, 28 and 56** ^**a**^ **: number of dogs/number of dogs per group)**				
**Clinical sign**	**Day −4**	**Day 28**	**Day 56**	**Day 84**
Erythematous patches	87.5% (7/8)	50% (4/8)	0% (0/8)	0% (0/7)^b^
Crusts, casts or scales	100% (8/8)	100% (8/8)	37.5% (3/8)	42.9% (3/7)^b^

^a^Skin assessments were performed before treatment.

^b^On day 59 one dog had to be euthanized due to the presence of a malign tumour in the stomach.

**Figure 1:**
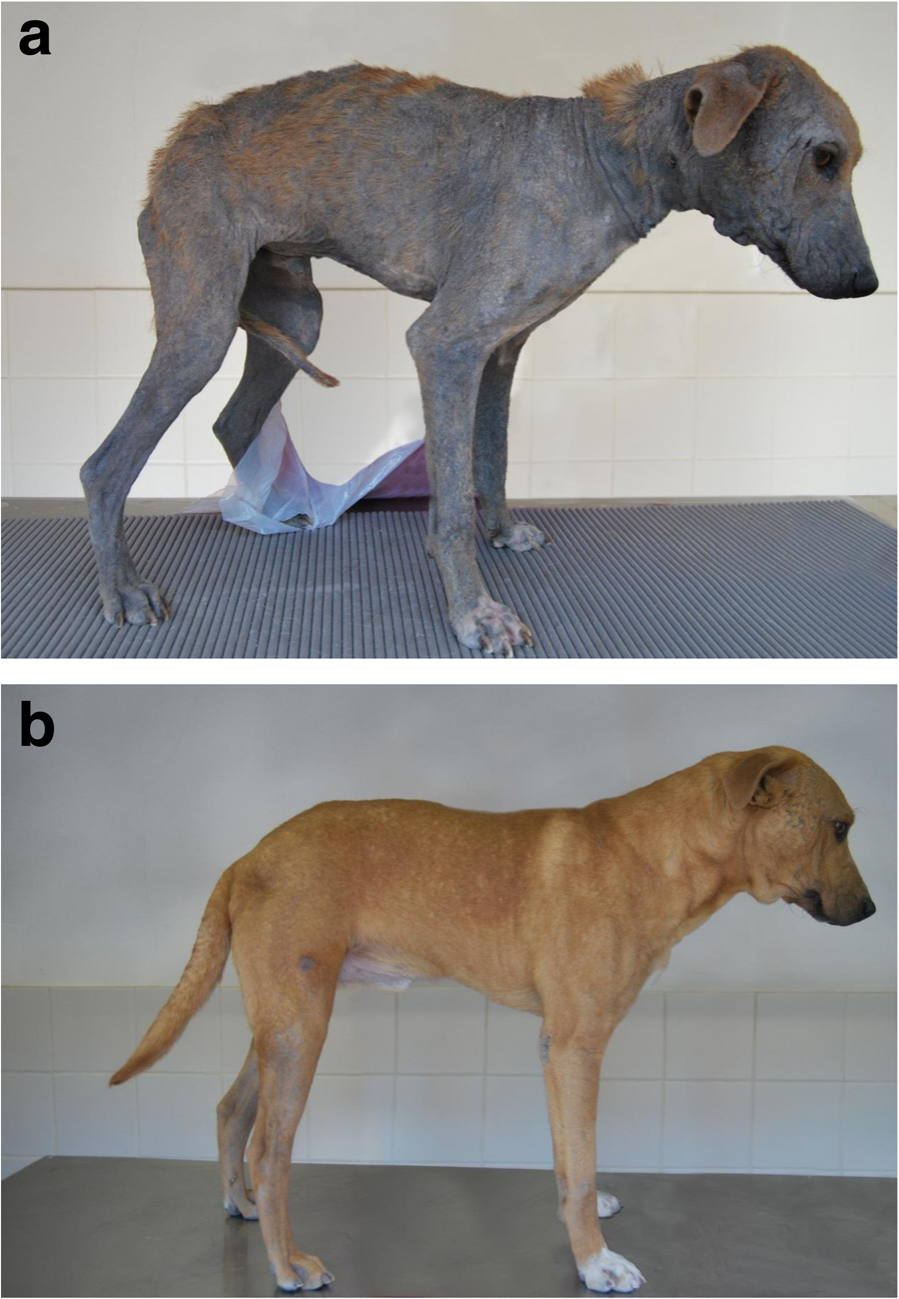
**Example of hair re-growth in a dog suffering from generalized demodicosis pre-treatment (a) and 12 weeks after initiation of treatment (b).**

Hair re-growth compared to the proportion of the body area covered by hair prior to treatment is summarized in Table 3. By Days 56 and 84 after initiation of treatment, hair re-growth on the majority of dogs in both groups exceeded the hair-coat of the dogs by 90% compared with the pre-treatment assessment (Figure 1).

**Table 3:** **Hair re-growth on dogs with generalized demodectic mange after treatment with Bravecto**™ **or Advocate®**

**Study day**	**Estimated percent hair re-growth** ^**a**^					
	**Bravecto** ^**™b**^ **(number of dogs/number of dogs per group)**			**Advocate®** ^**c**^ **(number of dogs/number of dogs per group)**		
	**0-50%**	**50-90%**	**>90%**	**0-50%**	**50-90%**	**>90%**
**28**	3/8	1/8	4/8	6/8	1/8	1/8
**56**	0/8	1/8	7/8	0/8	1/8	7/8
**84**	0/8	1/8	7/8	0/7^d^	1/7^d^	6/7^d^

^a^Percentage hair re-growth defined as estimated percentage of hair cover growth compared to baseline total hairless area as assessed prior to veterinary product administration.

^b^Dogs were treated once orally on Day 0.

^c^Dogs were treated topically on Day 0, on Day 28 and again on Day 56. Hair assessments were performed before treatment.

^d^On day 59 one dog had to be euthanized due to the presence of a malign tumour in the stomach.

The body weight of every dog increased similarly in both groups during the study period.

## Discussion

A single oral administration of Bravecto™ chewable tablets to dogs with naturally acquired generalized demodicosis resulted in significantly lower mean mite counts 56 and 84 days after treatment and a correspondingly greater efficacy at each time period compared to three successive treatments with Advocate® at an interval of 28 days. No mites were present in the skin scrapings of the dogs 56 or 84 days after treatment with Bravecto^TM^ chewable tablets, while a few mites were still present in skin scrapings 56 (mite average: 18.5) and 84 (mite average: 25.6) days after the initial treatment with Advocate®.

Reduction of mite counts was consistent with reduction in the extent and severity of the skin changes. Both groups of dogs showed resolution of the associated dermatologic signs with steady improvement over the 12 week treatment period. More Bravecto™ treated dogs had resolution of crusts, casts and scales and showed hair re-growth, while none of the Advocate® treated dogs had erythema at the assessment time point Day 84.

A problem frequently encountered with the treatment of demodicosis in dogs is the inability to ensure that a dog is absolutely free from mites after completion of a specific treatment regimen and re-infestation can be detected months after a treatment that was initially considered to be successful [15,16]. The present results are, however, encouraging that the administration of Bravecto™ chewable tablets offers the potential to provide sustained control of demodex mite infestations in susceptible dogs for at least three months after a single treatment.

Owner compliance can be an important factor in treatment success when multiple doses of a treatment spread over a long period of time are required in order to achieve a satisfactory outcome. Bravecto™ administered once as chewable tablets is not only effective against *Demodex* spp. mites on dogs but remains effective for 12 weeks following treatment. Moreover, it is effective for the same period of time against ticks and fleas that may concomitantly infest these animals [20,21]. Therefore, the single administration may help to reduce the risk of treatment failure as a consequence of poor owner compliance with treatment recommendations.

Bravecto™ chewable tablets have proved to be safe for use in dogs at five times the recommended therapeutic dose [26]. It is also safe to use in breeding, pregnant and lactating dogs. With this in mind it could prove to be an effective prophylactic intervention against the transmission of *Demodex* spp. mites from a post-parturient bitch to her new-born pups aiding in the prevention and control of demodicosis in all its forms on the following generation of dogs.

## Conclusions

Single oral administration of Bravecto™ chewable tablets is highly effective against generalized demodicosis, with no mites detectable at 56 and 84 days following treatment. In comparison, Advocate®, administered three times at 28-day intervals, is also highly effective against generalized demodicosis, but most dogs still harboured mites at all assessment time points. Both treatments resulted in a marked reduction of skin lesions and increase of hair re-growth 12 weeks after the initial treatment.
